# Moebius Syndrome with Hypoglossal Palsy, Syndactyly, Brachydactyly, and Anisometropic Amblyopia

**DOI:** 10.7759/cureus.2334

**Published:** 2018-03-16

**Authors:** Muhammad Hassaan Ali, Samreen Jamal, Muhammad Ather Rashid, Usman Javaid, Nadeem Hafeez Butt

**Affiliations:** 1 Department of Ophthalmology, UCLA Stein Eye Institute; 2 Ophthalmology, King Edward Medical University(KEMU)/Mayo Hospital Lahore,Pakistan; 3 Ophthalmology, Jinnah Hospital Lahore (JHL)/Allama Iqbal Medical College (AIMC), Lahore, Pakistan.

**Keywords:** lagophthalmos, congenital facial palsy, moebius syndrome, syndactyly, brachydactyly, amblyopia

## Abstract

Moebius syndrome is a rare cause of congenital facial and abducens palsy.^ ^It is sometimes associated with musculoskeletal abnormalities and other cranial nerve palsies. Genetics and ischemic insults to the fetus are considered to be the cause of this syndrome. We report here a 12-year-old female patient who was presented to us with poor cosmesis of her face, with associated decreased vision and lagophthalmos in her left eye. She didn’t have any signs of exposure keratopathy in the affected eye. Her best-corrected vision was 20/20 and 20/60 in right and left eyes respectively. The cause of decreased vision in her left eye was found to be anisometropic amblyopia, due to asymmetric hyperopic astigmatism in her eyes. She did not report diplopia in any gaze position. Examination of her cranial nerve revealed left facial, abducens, and hypoglossal nerve palsy, leading us to the diagnosis of Moebius syndrome. Apart from that, she had syndactyly in one of her hands, and brachydactyly in both. Since the eyes were straight in their primary position, no surgical intervention was carried out for her lagophthalmos, which was measured to be only 2 mm. This was to prevent any post-operative iatrogenic ptosis. The condition requires a multidisciplinary approach involving the opinions of a neuro-ophthalmologist, strabismologist, and oculoplastics for the management of the complications associated with the disease.

## Introduction

Moebius syndrome is one of the very rare congenital causes of facial palsy. It was first described by a German neurologist, Paul Moebius, in 1892 [1]. Initially, it was believed to comprise of bilateral facial and abducens nerve palsy but, later on, cases with unilateral facial and abducens involvement were also included in the spectrum of this disease [2]. Various congenital deformities have been reported with the syndrome, which has led some authors to declare it as a syndrome due to abnormal development of the hind-brain [3]. Various musculoskeletal abnormalities are often reported with the condition. Here, we report a case of Moebius syndrome with unilateral facial and abducens involvement, with syndactyly, brachydactyly, and anisometropic amblyopia.

## Case presentation

Our patient was a 12-year-old female who presented to us with “poor cosmesis” of her face, decreased vision, and an inability to close her left eye since birth. Initially, the parents noticed that the child had multiple episodes of recurrent watering in her left eye during the first five months of her life. But later on, they clearly noticed that she could not close her left eye and had an unusual facial appearance while smiling or crying.

The mother of the patient was 28 years of age at the time of birth of the patient. The mother did not have any history of antenatal infection or fever. She had not used any medications or supplements during pregnancy as well. The patient was born by normal vaginal delivery without any complications. There were three siblings of the patient who were all healthy without any medical illness or any similar abnormality in the family.

Ophthalmic examination of the patient revealed a best corrected visual acuity (BCVA) of 20/20 and 20/60 in her right and left eyes, respectively (Table 1). The cause of her decreased vision in her left eye turned out to be anisometropic amblyopia, due to asymmetric hyperopia and astigmatism in her eyes. The patient gave a history of patching her right eye for one year for amblyopia treatment at the age of 10 years, but mentioned that it did not significantly improve her vision.

**Table 1:** Detailed ophthalmic examination of the patient

Parameter	Right Eye	Left Eye
Best Corrected Vision	20/20	20/60
Refraction	+3.25 / -1.50 x 10	+5.75 / -2.25 x 180
Color Vision	Normal	Normal
Adnexa	Normal	Lagophthalmos
Cornea	Clear	Clear
Pupil	PERRLA	PERRLA
Anterior Chamber	Deep and quiet	Deep and quiet
Lens	Clear, phakic	Clear, phakic
Fundus	CDR = 0.3, healthy disc and macula and vessels	CDR = 0.4, healthy disc and macula and vessels
CDR = Cup to Disc Ratio; PERRLA = Pupils Equal Round Reactive to Light and Accommodation		

**Figure 1:**
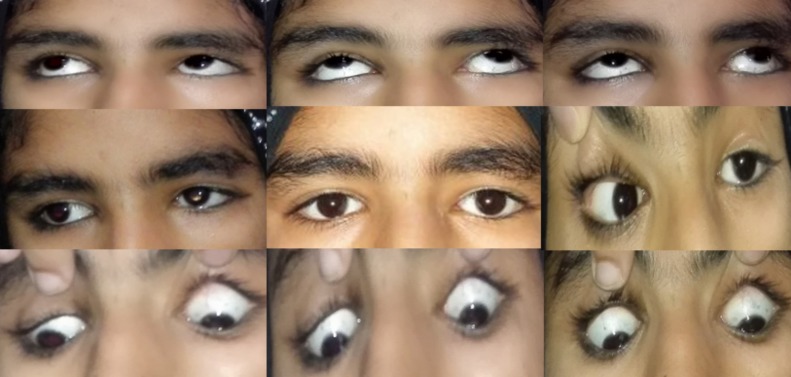
Extraocular motility showing left abducens palsy leading to absent abduction on left side

The patient had lagophthalmos in her left eye. On complete closure of her left eye, the patient had a space measuring 3 mm between her upper and lower eyelids. The corneal sensitivity was normal, symmetric, and intact in both the eyes. Slit-lamp examination revealed no corneal subepithelial punctate erosions, abrasions or any other sign of exposure keratopathy in the affected eye. The patient exhibited normal Bell’s phenomenon in both of her eyes. The extraocular motility testing of the patient showed a normal central position of the eyes in primary gaze. However, there was a left lateral rectus palsy leading to an absent abduction in the left eye (Figure 1). The rest of the movements in all gaze positions were intact. The patient did not have diplopia or ocular pain in any of the gaze positions.

**Figure 2:**
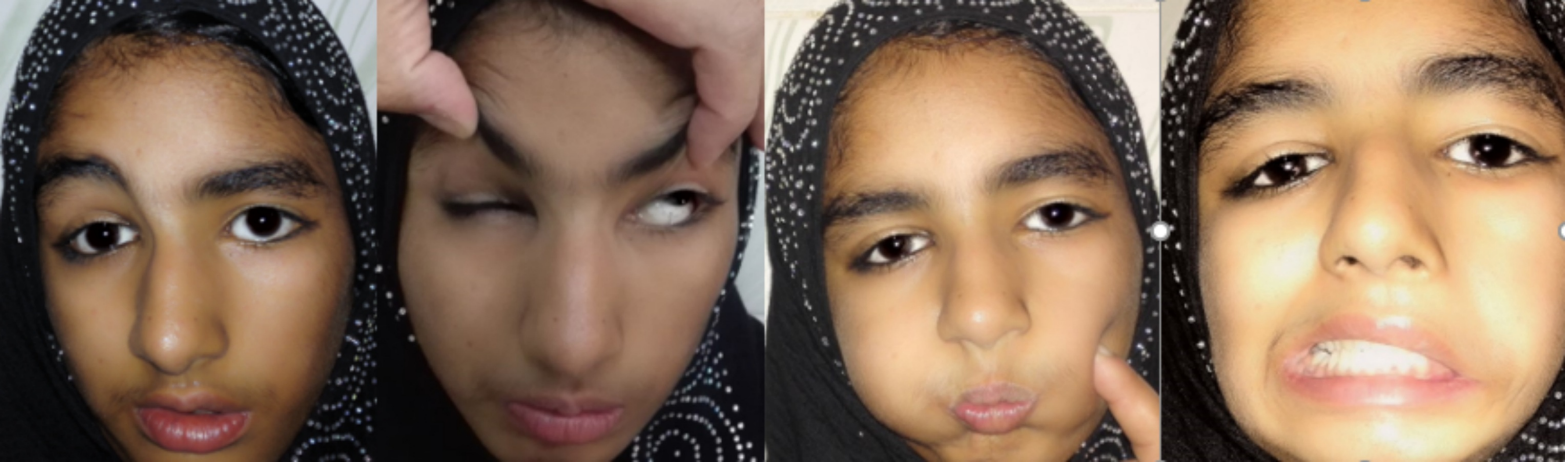
Facial nerve examination of the patient Pictures reproduced here with the permission of the guardians.

**Figure 3:**
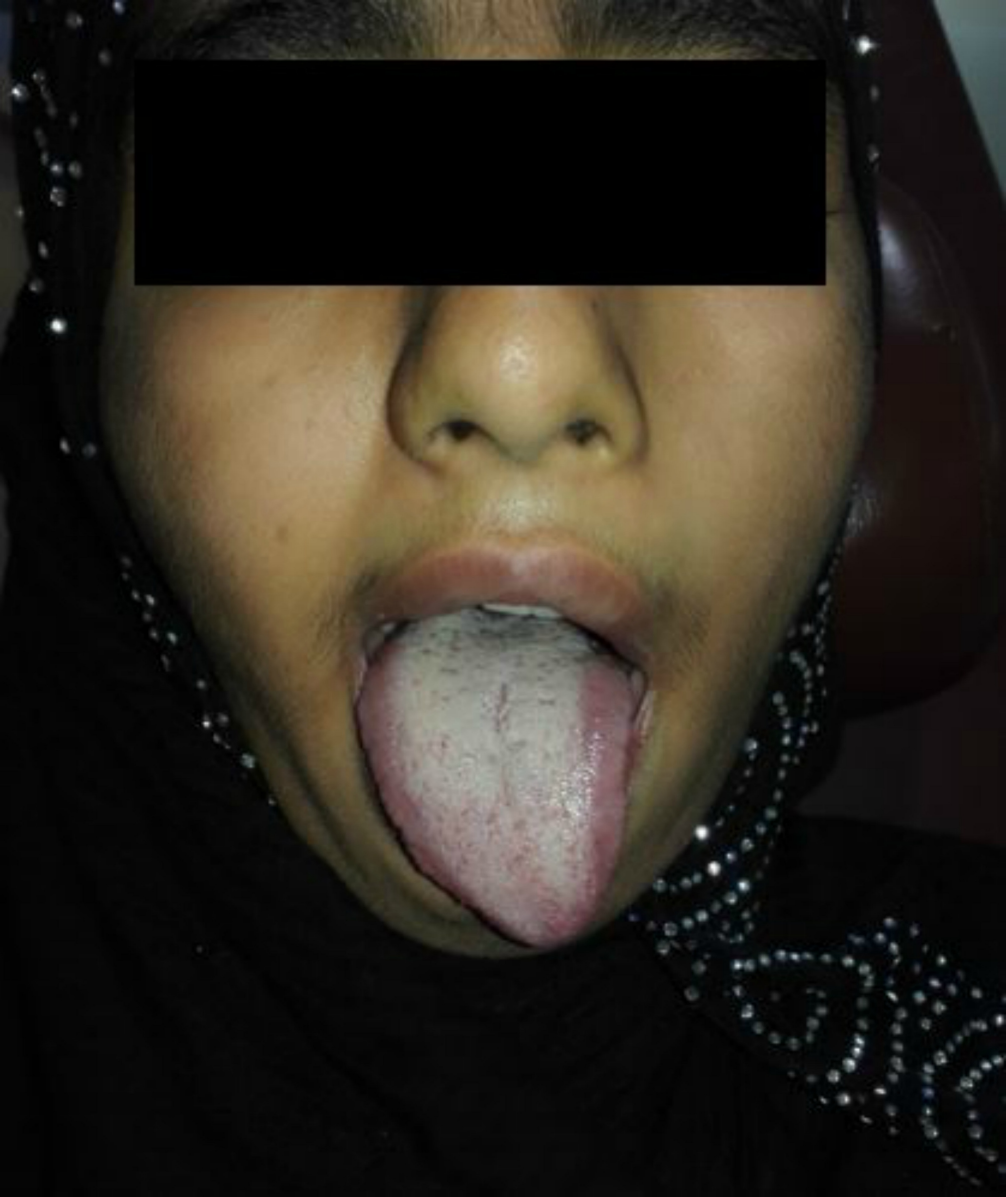
Left hypoglossal palsy with deviation of tongue to left side

Cranial nerve examination showed left facial palsy (Figure 2). When asked to crease up the forehead, no furrows were observed on the left side. She could not keep her left eye closed against resistance, and there was deviation of the angle of her mouth to the right side when asked to reveal her teeth. When asked to close her eyes, there was an incomplete closure of the left eye but due to normal Bell’s phenomenon, both the eyes rolled up-and-out, leaving no part of cornea exposed. When asked to protrude her tongue, there was deviation of the tongue to the left side, pointing towards left hypoglossal nerve palsy (Figure 3). Rest of the central nervous system (CNS) and cranial nerve examinations were normal. Her hearing was intact as well. She did not have any other abnormality in cardiovascular, gastrointestinal, urinary, and reproductive systems.

**Figure 4:**
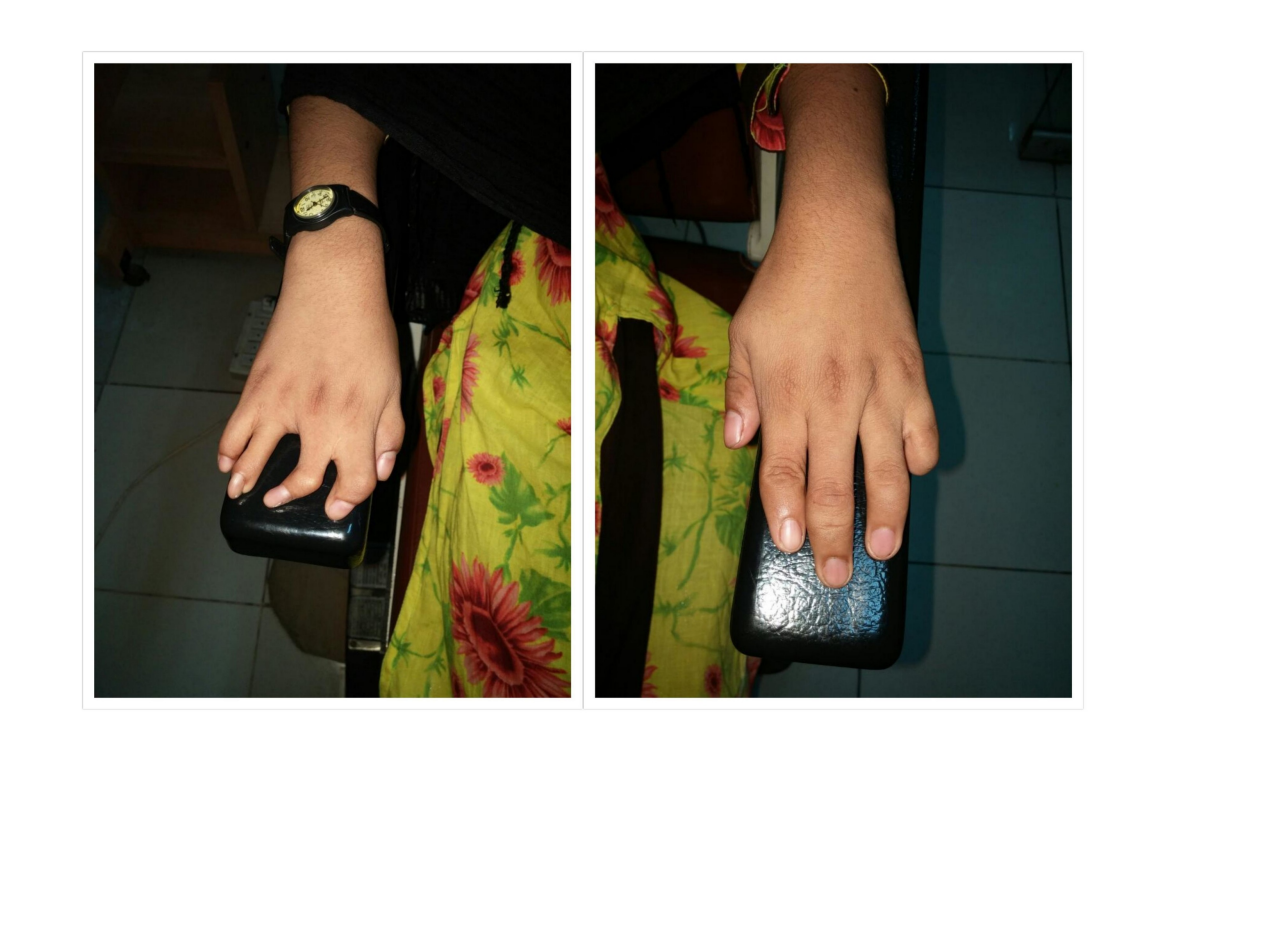
Musculoskeletal anomalies released syndactyly in right hand, auto-amputation of little finger in left hand, and brachydactyly in both hands.

**Figure 5:**
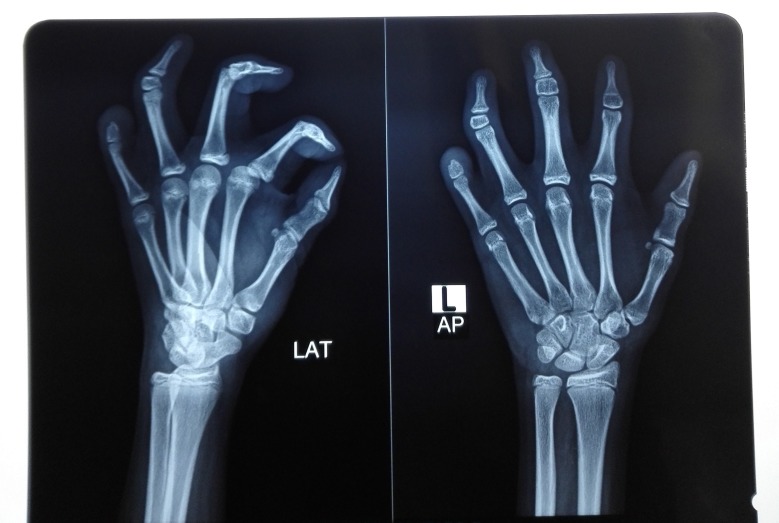
X-ray images of the skeletal abnormalities

The child had associated musculoskeletal deformities as well. She had syndactyly in one of her hands, and brachydactyly in both. She had already been operated to release her syndactyly at the age of six years (Figures 4-5). 

Her various laboratory investigations revealed a healthy profile. The x-rays of her hands revealed a rudimentary middle-phalanx in most of her fingers, while an absent distal-phalanx in the little finger of her left hand was leading to brachydactyly. The magnetic resonance imaging (MRI) of her brain also showed normal cranial nerve complexes with no signs of cranial nerve nuclei hypoplasia and no other gross CNS pathology. 

## Discussion

We hereby report a case of Moebius syndrome with unilateral facial and abducens palsy, with brachydactyly, syndactyly, and anisometropic amblyopia. Our patient fulfilled multiple diagnostic criteria for the diagnosis of Moebius syndrome. Usually, bilateral facial and abducens palsy are observed in this disease, but unilateral cases have also been included in the spectrum of this disease, which is rarely found. The common musculoskeletal abnormalities and brachial malformations seen with the syndrome include short tongue, short jaw, syndactyly, brachydactyly, limb deformities, club feet, and cleft palate [4].

The patients suffering from this rare disease present with mask-like faces, deviation of angle of the mouth especially during smiling or frowning, lagophthalmos with or without exposure keratopathy, inability to abduct their eye(s), esotropia in the affected eye, horizontal gaze palsies but intact and normal vertical gaze, and associated various congenital deformities [5]. A characteristic subset of patients with Moebius syndrome present with pectoral hypoplasia with underdevelopment of ipsilateral breast, which has been separately named as Poland syndrome [6].

So far, two pathophysiologic mechanisms are considered to be the cause of this syndrome: 1) genetic causes and 2) ischemic injury to the fetus during pregnancy, with the latter being the more common phenomenon [7]. Various loci have been described whose mutations produce the constellation of symptoms of the disease. These include 13q12.2-13.16,17 and 1p2214,157. Most cases of the disease are known to occur sporadically, but rare cases of autosomal dominant, autosomal recessive, and X-linked recessive have been reported in the literature. The other proposed mechanism is fetal ischemia, which impedes blood supply to the cranial nerve nuclei, leading to their hypoplasia or atrophy. This may be detected later on by a magnetic resonance imaging (MRI) of the brain [8]. Some cases may present without the involvement of the cranial nerve nuclei and may occur due to ischemia to various muscle groups and peripheral nerves. Our case appeared to be a sporadic case due to lack of history of such disease in the family. Since the MRI was normal, we proposed that the peripheral nerve ischemia is the cause of this disease in our patient.

Some environmental factors have also been reported to be the cause of this condition. An established teratogenic factor is the use of misoprostol, a prostaglandin analog used as an abortifacient and to treat gastric ulceration during pregnancy. It causes fetal ischemia and produces the disease. Other environmental toxins include cocaine, ergotamine, benzodiazepines, thalidomide, and zonisamide which when used during pregnancy, has been reported to cause this disease [9].

The incomplete closure of the left upper lid led to the impression of moderately severe facial nerve dysfunction in this patient. The amblyopia present in this patient was explained by the refractive asymmetry between the two eyes, especially hyperopia and astigmatism. Despite lagophthalmos, there was no corneal involvement in the affected eye, pointing towards good Bell’s phenomenon in the eye. We considered upper lid gold weight implantation in this patient. The residual lagophthalmos after upper lid gold weight implant can measure up to 2 mm [10]. Since there was no corneal involvement in this patient, the eyes were straight in the primary position, and the patient’s lagophthalmos measured only 2 mm, we decided not to do any surgical intervention in the case to prevent post-operative iatrogenic ptosis. The patient was advised medical treatment with lubricant, ointments, drops, and was counseled to report to the hospital in case of redness, irritation, burning in her left eye.

The differential diagnosis for Moebius syndrome include: 1) Melkerrson Rosenthal syndrome that presents with characteristic triad of congenital facial palsy, tongue fissuring and lip swelling; 2) Poland syndrome with congenital facial and abducens nerve palsy with ipsilateral pectoralis muscle hypoplasia; 3) hereditary congenital facial palsy (HCFP) with isolated facial palsy without abducens involvement; and 4) Duane retraction syndrome that presents with limited abduction or adduction of the eye, inward retraction of the eyeball and shortening of palpebral fissure on horizontal eye movements [5-6,8].

The treatment options for lagophthalmos due to congenital facial palsy include [9]:

• Medical management: lubricant ointments, eye drops, taping to prevent exposure keratopathy

• Partial or complete tarsorrhaphy

• Other mechanical techniques for reanimating lid closure including:

• Palpebral springs

• Encircling the upper and lower eyelids with silicone or fascia lata, and temporalis muscle transfer

• Most recently, upper-eyelid gold weight implant for gravity assisted treatment of the functional defect of lagophthalmos resulting from facial paralysis [10].

The treatment for the condition is symptomatic and according to the complaints of the patient. The patient should be managed by a multidisciplinary team consisting of a neurologist, ophthalmologist, pediatrician, plastic surgeon, and physiotherapists. The patients and their families should be well-counselled regarding the complications and morbidity associated with the disease. To the best of our knowledge, this is the first case reported on the subject from Pakistan.

## Conclusions

Moebius syndrome is a very rare cause of congenital facial nerve palsy in children who present with lagophthalmos and other features of facial nerve palsy. Neurological examination should rule out involvement of other cranial nerves especially abducens and hypoglossal nerve. Associated musculoskeletal anomalies like brachydactyly and syndactyly should always be screened for. The condition demands a team-based approach including a strabismologist, neurologist, and oculoplastic surgeon besides detailed physical examination by a pediatrician. The benefits of lagophthalmos treatment should always be weighed against the risk of iatrogenic ptosis and should be tailored according to the cosmetic demands of the patient.
